# Incorporating priors for EEG source imaging and connectivity analysis

**DOI:** 10.3389/fnins.2015.00284

**Published:** 2015-08-18

**Authors:** Xu Lei, Taoyu Wu, Pedro A. Valdes-Sosa

**Affiliations:** ^1^Sleep and NeuroImaging Center, Faculty of Psychology, Southwest UniversityChongqing, China; ^2^Key Laboratory of Cognition and Personality, Ministry of EducationChongqing, China; ^3^Key Laboratory for NeuroInformation of Ministry of Education, School of Life Science and Technology, University of Electronic Science and Technology of ChinaChengdu, China; ^4^Cuban Neuroscience CenterCubanacan, Playa, Cuba

**Keywords:** EEG source imaging, multimodality, brain network, EEG-fMRI

## Abstract

Electroencephalography source imaging (ESI) is a useful technique to localize the generators from a given scalp electric measurement and to investigate the temporal dynamics of the large-scale neural circuits. By introducing reasonable priors from other modalities, ESI reveals the most probable sources and communication structures at every moment in time. Here, we review the available priors from such techniques as magnetic resonance imaging (MRI), functional MRI (fMRI), and positron emission tomography (PET). The modality's specific contribution is analyzed from the perspective of source reconstruction. For spatial priors, EEG-correlated fMRI, temporally coherent networks (TCNs) and resting-state fMRI are systematically introduced in the ESI. Moreover, the fiber tracking (diffusion tensor imaging, DTI) and neuro-stimulation techniques (transcranial magnetic stimulation, TMS) are also introduced as the potential priors, which can help to draw inferences about the neuroelectric connectivity in the source space. We conclude that combining EEG source imaging with other complementary modalities is a promising approach toward the study of brain networks in cognitive and clinical neurosciences.

## Introduction

Electroencephalography (EEG) measures the brain's electric fields that are quantifiable at various scalp sites. Its main contributor is the extra- and intra-cellular electric currents associated with neuronal activity. Recently, EEG has been recognized as a useful technique to non-invasive study of brain dynamics. Here are some reasons: First, the EEG system is easy to be manipulated and cheap to be set up. Second, EEG can take direct measurement of the real-time response from neural activity without delay. Third, EEG has the high temporal resolution that is suitable to investigate dynamic brain activation during various cognitive tasks. Fourth, the modern EEG has greatly improved in its spatial resolution with the increased number of electrodes. Advantages above make EEG a widely utilized technique in cognitive and clinical neurosciences.

The reconstruction of electrophysiological activity in the cortex based on scalp EEG is a typical inverse problem (Helmholtz, 1853). Due to a large number of unknown parameters comparing to the number of scalp electrodes, the spatial location of the neuronal sources of the scalp recorded activity cannot be conclusively determined, i.e., the inverse problem has no unique solution (Baillet et al., 2001). Infinite configurations of neuronal sources can lead to the same scalp potential measurement. A priori assumptions based on physiological and biophysical knowledge have to be incorporated to reduce the number of unknown parameters and finally solve the problem. The rationality of the related knowledge determines the correctness of the source imaging. Currently, the available priors in EEG source imaging (ESI) gain from magnetic resonance imaging (MRI), functional MRI (fMRI), positron emission tomography (PET), diffusion tensor imaging (DTI), transcranial magnetic stimulation (TMS), etc.

**Figure 1:**
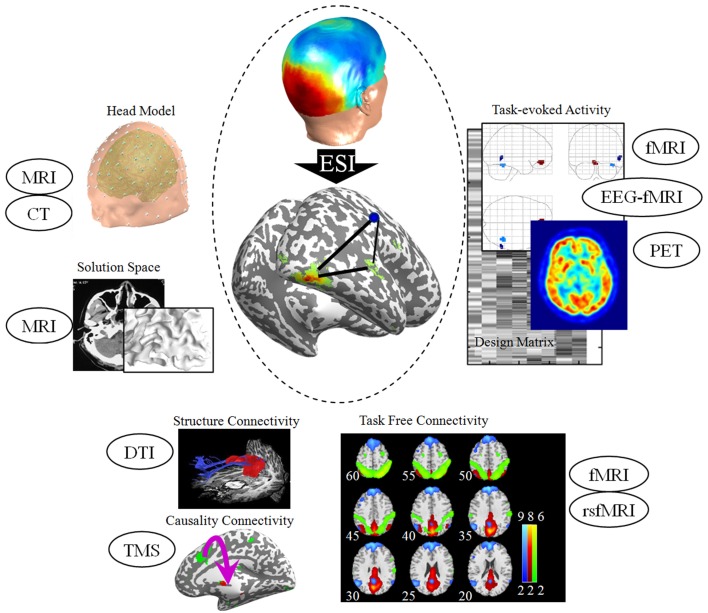
**Priors for EEG source imaging**. The anatomical and structural information derived from MRI and CT advances the precision of the head model. Spatial priors in ESI include task-evoked activity derived from fMRI, EEG-correlated fMRI and PET. A recent extension of spatial prior is task-free connectivity derived from resting-state fMRI. For connectivity analysis, fiber tracking derived from DTI and neuro-stimulation techniques such as TMS advance the inference of neuroelectric network in the cortex space. The TMS alters cortical excitability, effective connectivity, and oscillatory tuning of a given cortical area, and hence provides both spatial and connectivity priors for ESI. All the sources are from our unpublished experimental data. MRI, magnetic resonance imaging; CT, computed tomography; EEG, Electroencephalography; ESI, EEG source imaging; fMRI, functional MRI; PET, positron emission tomography; DTI, diffusion tensor imaging; TMS, transcranial magnetic stimulation.

Algorithms for ESI have been rapidly evolved into many directions over the last 20 years: from a single prior (Dale et al., 2000) to multiple priors (Friston et al., 2008); from the task-evoked activity (Henson et al., 2010) to the task-free connectivity (Lei et al., 2011b); from the asymmetric spatial constraint to the multimodal fusion (Baillet et al., 2001). Realistic head models based on detailed anatomical information that derived from the structural neuroimaging have improved the precision of forward model. The spatial prior from functional neuroimaging has advanced the accuracy of source localization. The precision of source localization has made significant progress in recent years, allowing real-time imaging of neural activity in the brain directly and non-invasively. As illustrated in Figure 1, many experimental and clinical studies have utilized diverse priors from other modalities and have validated their correctness of the localization by some direct evidence such as intracranial recordings.

In this article, we reviewed the special contribution of each modality to ESI, containing such techniques as MRI, fMRI, PET, DTI, and TMS. First, we will discuss the mathematic frameworks in combining priors. Then, we have a systematic analysis of the spatial priors and it mainly includes EEG-correlated fMRI, temporally coherent networks (TCNs) and resting-state fMRI. Moreover, as functional and causality connectivity become a central topic in the neuroimaging community, the fiber tracking and neuro-stimulation techniques, corresponding to DTI and TMS respectively, are also introduced as the priors, which can help to draw inferences about the neuroelectric connectivity. More than providing definitive answers, we aim to identify important open issues in the quest of incorporating priors from other modalities into ESI.

## Mathematical models to incorporating priors

Here we only introduced two frameworks which were widely utilized to incorporate priors into ESI. Penalty function employs different types of norm and weight matrices to incorporate priors from other modalities. Alternatively, Bayesian framework considers prior information as a prior probability and computes the posterior probability of source distribution.

### Penalty function

Penalty function imposes constraints on the source activity *g* in a multiple-penalized model. The corresponding ESI estimation is stated as (Valdés-Sosa et al., 2009b):

(1) $$\hat{g}=\arg\underset{g}{\min}({\left\|Y-Lg\right\|}_{2}^{2}+\sum_{i\;=\;1}^{k}\lambda_{i}{\left\|W_{i}g\right\|}_{p})$$

where *ĝ* ∈ *R*^*d*×*s*^ is the unknown source dynamics for *d* dipoles. *Y* ∈ *R*^*n*×*s*^ is the EEG recording with *n* sensors and *s* samples, and *L* ∈ *R*^*n*×*d*^ is the known lead-field matrix. ‖*W*_*i*_*g*‖_*p*_ is a penalization function with *l*_*p*_-norm, *W*_*i*_ is a weight matrix imposing different type of constraint and λ_*i*_ is a regularization parameter determining the relative importance of each constraint. This model includes all prior information through the *k* penalty functions. As both the types of norm and the weight matrix are selectable, the penalized model is flexible to incorporate different constraints.

Several forms of *W* have been proposed for EEG source imaging. Obviously, the identity matrix produces the classical minimum norm model (MNM) (Hämäläinen and Ilmoniemi, 1994). Because the gain matrix introduces an intrinsic bias that favors solutions in close proximity to the sensors in MNM reconstructions. While the normalized MNM has reduced this problem through bias adjustment (Jeffs et al., 1987) though it suffers from a depth bias *per se* (Lucka et al., 2012). For normalized MNM, *W* is a matrix with diagonal elements equal to the norm of the corresponding column of the gain matrix *L*. *W* can further have a spatial derivative of the image of first order or Laplacian form, and the latter corresponds to the low-resolution electromagnetic tomography algorithm (LORETA) (Pascual-Marqui et al., 1994). In an effort to reduce the spatial blurring of LORETA, the focal underdetermined system solution (FOCUSS) algorithm emphasizes strong sources while decreases weak ones by iteratively updating *W* (Gorodnitsky and Rao, 1997). In application of multimodality, *W* is a diagonal matrix with elements equal to the estimated source power at that location, which may be the task-evoked activation evaluated in each voxel from fMRI or PET.

The type of norms is another selectable item to restrain the source distribution. In most study *p* = 2, where the symbol ‖·‖_2_ denotes *l*_2_-norm. The estimators are explicit and linear for this quadratic function, offering smooth solutions. Recently, there have been advances in solving the EEG inverse problem by a sparse and local solution that highlight the use of non-convex (non-quadratic) penalty functions (Valdés-Sosa et al., 2009b). A variety of methods have been proposed such as *l*_1_ norm solution [i.e., the least absolute shrinkage selection operator (LASSO)] (Silva et al., 2004), and *l*_p_ norm iterative sparse source (LPISS) (Xu et al., 2007). Recently, an *l*_0_ constrained penalty function is introduced in the solution space sparse coding optimization (3SCO), which codes the solution space with some particles. The particle-coded space is compressed by the evolution of particle swarm optimization algorithm (Xu et al., 2010).

### Bayesian framework

Bayesian allows a priori assumption of EEG source to be explicitly quantified by using postulated prior distributions. The types of prior incorporated in Bayesian include information on the neural current (David et al., 2006), the combined spatial and temporal constraints (Trujillo-Barreto et al., 2004), as well as the sparse nature of the sources (Friston et al., 2008). Recent studies have shown that a promising tool for reliable estimation of EEG sources is parametric empirical Bayesian (PEB) (Phillips et al., 2005; Henson et al., 2010), which is:

(2) $$\begin{array}{rcl} Y & = & Lg+\varepsilon_{1}\;\varepsilon_{1}\sim N\left(0,T,C_{1}\right)\\ g & = & 0+\varepsilon_{2}\;\varepsilon_{2}\sim N\left(0,T,C_{2}\right), \end{array}$$

where ε_1_ and ε_2_, both obeying multivariate Gaussian distributions, representing random fluctuations in sensor and source spaces, respectively. The temporal correlations are denoted by *T*, and the spatial covariance of ε_2_ are mixtures of covariance components (CCs), i.e.,

(3) $$\begin{array}{r} C_{2}=\overset{k}{\underset{i=1}{\sum }}\gamma_{i}V_{i}, \end{array}$$

where $\gamma \equiv {\left[\gamma_{1},\gamma_{2},\ldots ,\gamma_{k}\right]}^{T}$ is a vector of *k* non-negative hyperparameters that control the relative contribution of each CC, *V*_*i*_. The components set, *V* = {*V*_1_, *V*_2_, …, *V*_*k*_}, provides an extremely flexible framework to incorporate priors from other modalities. The number of components could range from one (such as *V* = {*I*} in the classical MNM), to hundreds [such as multiple sparse prior (MSP), in which each component accounts for a certain compact spatial support; (Friston et al., 2008)]. In addition to the mathematical priors above, the task-evoked activity derived from fMRI is widely considered in EEG source imaging. For example, the statistical parametric map (SPM) derived from a common general linear model (GLM) of fMRI was adopted in an EEG source imaging method and it was named as dynamic SPM (dSPM) (Dale et al., 2000). We will discuss the form of CCs in Section Temporally Coherent Networks. Once the form of spatial correlations of the sensor noises and the lead-field are given, the model would be determined by the composition of the empirical priors related to the sources. The hyperparameters γ are akin to the standard regularization parameters in ill-posed problems, and can be estimated with various Bayesian inferences (Friston et al., 2008).

### Relations between bayesian and penalty function

**Figure 2:**
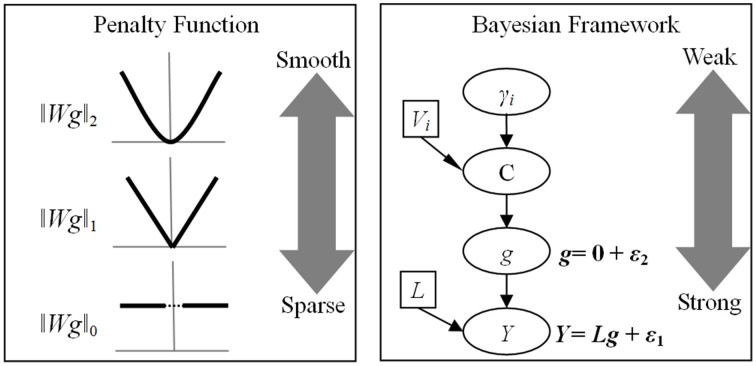
**Relationship between penalty function and Bayesian model**. The advantages of penalty function are the configurable norm and the weight matrix. In contrast, Bayesian model has a hierarchical structure, which yield different influence on the final solution. ‖*Wg*‖_*p*_ is a penalization function with *l*_p_-norm, *W*_*i*_ is a weight matrix imposing different type of constraint and *g* is the unknown source activity. While most studies set *p* = 2 to employ *l*_2_-norm, recently developments proposed both *l*_1_ and *l*_0_ norms to obtain the sparse solutions. *Y* is the EEG recording and *L* is the known lead-field matrix. ε_1_ and ε_2_ obey multivariate Gaussian distributions, representing random fluctuations in sensor and source spaces, respectively. The spatial covariance of *g*, C, is mixtures of covariance components (*V*_*i*_) and hyperparameters (γ_*i*_).

Though aforementioned frameworks have different forms, the core structures are similar and can be easily translated to each other. The advantage of penalized model is that both the types of norm and the weight matrix are configurable (Figure 2). The choice between sparse and smooth is valuable because it is easy to design an experiment with specific penalized model. A balance can also be achieved by combining with multiple penalty functions in a single objective function (Lei et al., 2009). In this way, a family of models combining *l*_1_ norm and *l*_2_ norm describe a continuous transition of one type of ESI to the other (Vega-Hernandez et al., 2008). The functionally significant solution may be identified with the blend of smoothness and sparseness.

The superiority of Bayesian model is its hierarchical structure (Figure 2). In this model, the lower level has direct and strong influence on the solution, while the higher level only influences its adjacent levels and has weak influence on the final solution. The prior distribution and the inference procedure that are subsequently adopted have many choices, the implementations of Bayesian model have led to a large number of algorithms with seemingly very different properties.

In Bayesian model, if the prior distribution satisfies multivariable Gaussian distribution, it can be easily translate into a quadratic penalty function though transformation of logarithm. In fact, there are explicit connections between many established algorithms (Wipf and Nagarajan, 2009), including the LORETA, FOCUSS, minimum current estimation, restricted maximum likelihood, variational Bayes, the Laplace approximation, automatic relevance determination, and beamforming (Van Veen et al., 1997). The beamformer is a spatial-filtering approach filtering the EEG signal by different beams and it is based on lead-field matrix corresponding to some specific source points (Sekihara et al., 2001). Surprisingly, all of these methods can be formulated as particular cases of Bayesian distribution and optimization rules, making theoretical analyses and algorithmic improvements particularly relevant (Wipf and Nagarajan, 2009).

## Anatomical and structural information

The accuracy of ESI depends crucially on the head model and the solution space initiated to compute the forward problem. The related imaging modality contains computed tomography (CT) and MRI. The selected modalities influence the solution of ESI, according to their sensitivity to hard or soft tissues. For the majority of its practical applications, CT is usually not available as there is no sufficient indication to expose the subject/patient to the harmful ionizing radiation.

### The head models

The head models can determine the measurements on the scalp after locating the sources at the given positions. Both the electromagnetic (permeability and conductivity) and geometrical (shape) properties of the volume are considered in the head models. The simplest and still widely used head model is the spherical model. This model, with the uniform conductivity properties, allows for an analytical solution of the forward problem (Yao, 2000). Source localization accuracy is limited when using this model because the head is not spherical and also its conductivity is not spatially uniform (Michel et al., 2004). Incorporations of different conductivity parameters and consideration of local anisotropies in multi-shell spherical head models can ameliorate the accuracy to a certain degree. The overlapping-sphere head model was used to quickly calculate a more realistic head shape. The volume currents were employed to fit spherical head models for each individual electrode such that the head is modeled more realistically as some overlapping spheres, rather than a single sphere (Huang et al., 1999). Though the boundary element method (BEM) model has the similar accuracy with the overlapping sphere model, the latter is faster to compute.

Several simulations have shown that accurate lead field computation can only be achieved by using realistic volume conductor models, such as BEM model and the finite element method (FEM) model (Valdés-Hernández et al., 2009). Triangulations of the interfaces between compartments of equal isotropic conductivities are used in BEM model as a geometric model (Hämäläinen and Sarvas, 1989). In contrast, the FEM model tessellates the whole volume and considers the individual anisotropic conductivities of each element. It implies the FEM model can take skull-breaches and anisotropies into account, especially for the sources placed deeply in sulci (Van den Broek et al., 1998; Güllmar et al., 2010). However, the generation of realistic geometric models is not a trivial task, since it implies the accurate segmentation of MRI and stable tessellations of head compartments. In addition, the detailed anatomical information about tissue conductivities is rarely available. Because of the obvious complexity of realistic head models, efforts have been made over recent years to combine the computational efficiency of template model with more accurate descriptions of the individual head shape. An approximate model (AM) was proposed in the condition that individual's MRI is not available (Valdés-Hernández et al., 2009). The average models perform better than a random selected individual model or the usual average model in the MNI space. The AM seems a convenient tool in large and systematical clinical and research studies demanding EEG source localization, when MRI is unavailable.

### The solution space

Another important issue concerns the permissible solution space within which the sources are considered, i.e., the distribution of the fixed solution points. It is valuable not only for source reconstruction, but also to determine the coordinates of the sources in terms of Brodmann areas or Talairach coordinates. This information can further be used to draw conclusions about the activated neurophysiologic structures.

In the spherical head models, the whole volume within the sphere is assumed to be the solution space. Accordingly, deep structures, cerebellum, and ventricles are all acceptable. In realistic head models such as BEM model or FEM model, the MRI is taken into account to restrict the solution space to the structures where putative EEG sources can actually arise. The optimal selection of such a restricted solution space is based on the segmentation of the MRI into the gray and white matters. Because the interface between the gray and white matters forms a surface solution space of ESI, the orientation of the cortical source space surface can be considered to be an additional prior for ESI (Dale and Sereno, 1993; Lin et al., 2006). Based on the fair spatial resolutions provided by MRI, the sources of optical stimulus were revealed in the visual cortex at the individual level (Cottereau et al., 2011). The use of the individual MRI is in need of clinical cases when deformations or lesions are presented. Only by using the subject's own MRI can such lesion areas be excluded from the solution space.

## Spatial priors for ESI

Task-evoked activation identified by fMRI or PET is the widest utilized spatial prior in ESI. In this case, the same task was repeatedly conducted in another modality with higher spatial resolution. The identified activation regions provide individual information about the respond areas. Another spatial prior in clinical application is the structure abnormality because of lesion or infection. For example, the lesion area is accepted as the interictal epileptiform discharges (IED) point in local epilepsy. A recent study had investigated the concordance between EEG source detection and voxel-based morphometry (Betting et al., 2010). On average, the nearest voxels detected by these two methods are very close, suggesting that gray matter abnormalities are associated with focal IED.

### Statistical parametric map

Task-evoked activation obtained from the blood oxygen level dependent (BOLD) signal may be the most dominant spatial prior for ESI (Liu et al., 1998; Henson et al., 2010). In this approach, activation areas can be used either to initially seed dipoles within the activation regions found in the SPM for further dipole fittings (Ahlfors et al., 1999), or to constrain the spatial locations of the likely sources of EEG (Liu et al., 1998). For example, the visual components detected by EEG can be properly localized in according with the subject's fMRI retinotopic maps obtained from the subject *per se* (Di Russo et al., 2002). Another straightforward approach to implement the fMRI constraint is to threshold the SPM of fMRI and set the variance estimate to a non-zero value only at locations exceeding a certain threshold (George et al., 1995). However, simulation studies suggest that this approach is exceedingly sensitive to model misspecifications, in particular to the presence of generators of EEG signals that are invisible in fMRI (Liu et al., 1998). Using a partial fMRI constraint can greatly reduce the distorting effect on such potential misspecifications. This method is accomplished by setting the minimum a priori variance estimate to some finite, non-zero values (typically 10% of the maximum value) (Liu et al., 1998; Dale et al., 2000).

The task-evoked activation is easy to be accepted in the PEB framework of ESI (Phillips et al., 2002; Henson et al., 2010). For example, in Equation (3) the SPM of fMRI constructed a covariance component *V*_dSPM_ in dSPM (Dale et al., 2000). Set the off-diagonal terms to 0.0, while the diagonal terms of *V*_dSPM_ corresponding to supra-threshold nodes are assigned a weight of 1.0. A more complex model considers the discrepant contribution of each cluster or network in a single SPM (Henson et al., 2010; Lei et al., 2012a). Because activation map usually contains multiple clusters, they may have different contribution to ESI though all of them are significant activation areas in fMRI analyses. As a result, previous studies had conducted multiple spatial priors after classifying the clusters from the activation map, and then Bayesian modal selection was utilized to identify the most important clusters that are critical to generate scalp EEG (Henson et al., 2010; Lei et al., 2012a). In practice, the hierarchical structure in PEB considers a variety of fMRI information as priors (see Section Temporally Coherent Networks for more priors from fMRI).

### EEG-correlated fMRI

The simultaneous EEG-fMRI is a new technique to measure both the metabolic and electronic activities during mental processes. Here we introduced a method, named EEG-correlated fMRI, that can extract priors for ESI (Lei et al., 2011b). EEG-correlated fMRI investigates the hemodynamic changes associated with simultaneous electrophysiological activity. Assuming the regions identified in the simultaneous EEG-fMRI recording will reappear, one may expect to localize the sources via high-density EEG recording (Debener et al., 2006). We should note that the EEG in EEG-correlated fMRI is very different from the EEG used in ESI. In EEG-correlated fMRI, EEG acts like an oscilloscope with very fewer sensors than it is in ESI. Power of rhythm activity (Goldman et al., 2002) or amplitude of single-trial (Debener et al., 2006) are extracted from these limited sensors, and are further convolved with a canonical hemodynamic response function. The resulted signal is utilized as a hemodynamic predictor in a GLM. Assuming the degree of spatial concordance is good, one may exploit the high spatial resolution of fMRI to localize the generators of the high-density EEG signals based on a separated EEG session.

Previous EEG-correlated fMRI studies in patients with epilepsy often reveal that distributed patterns of BOLD signal changes are correlated with IED. Both the positive and negative BOLD cluster had equal contributions to ESI, and identified source at IED was closest to these clusters with maximal statistical significance (Vulliemoz et al., 2009). In addition, the region identified by EEG-correlated fMRI can further be used to initialize the contralateral sources, especially on the condition that the scalp EEG showed a bilateral distribution of the sources. Based on this assumption, we found a pair of bilateral sources at IED by modeling correlated sources in each hemisphere (Lei et al., 2011b).

ESI and EEG-correlated fMRI may use the same data if high-density EEG is conducted in a simultaneous fMRI recording. In this condition, EEG and fMRI can match with each other in the temporal and spatial domains. Moreover, a mutual beneficial iteration can be achieved in this condition. First, EEG-correlated fMRI provides the spatial priors for EEG source imaging. Then, the reconstructed source activity of ESI is further used as predictors of the BOLD signal changes. This method, named ESI-informed EEG-fMRI analysis, has more precise information about the amplitude of neural response, and may finally reveal the exact location of the BOLD signal changes (Vulliemoz et al., 2010). An focal epilepsy study proved its superiority when comparing with the conventional “event-related” designs based solely on the scalp EEG (Vulliemoz et al., 2010).

Though the cluster identified by EEG-correlated fMRI is valuable for EEG source imaging, this approach is rare utilized in application. In fact, the result integrated with the priors may be biased toward the activation map of EEG-correlated fMRI, while clinical application concerned more about the independent evidence from each modality. A reasonable alternation is to compare the sources derived separately from ESI and EEG-correlated fMRI. This method, named simultaneous ESI and EEG-fMRI analysis, accesses the degree of spatial concordance by using the EEG signals in parallel with both the ESI and the hemodynamic predictors of the BOLD signals. Simultaneous ESI and EEG-fMRI analysis has the potential to distinguish the areas of BOLD response related to the initiation of IED from the propagation areas (Vulliemoz et al., 2009, 2010). Generally, the fMRI-informed constraint on ESI is not recommended to map epileptic networks, unless a proper model comparison tool is included to assess the relevance of the BOLD clusters as ESI priors, for instance within a Bayesian framework for a posteriori assessment of the relevance of the fMRI constraints (Daunizeau et al., 2006).

### Temporally coherent networks

The brain exhibits TCNs both during the resting-state and the cognitive task. TCNs represent the interactions between different brain areas and involve numerous cortical and sub-cortical regions. In NEtwork based SOurce Imaging (NESOI), TCNs derived from task fMRI data are employed as the covariance priors of ESI (Lei et al., 2011b). As coherent analysis only underlines the time synchronization, both task-evoked and task-free activities are considered in NESOI. This is the main difference of NESOI when compared with other methods using traditional task-evoked priors from fMRI. This model also laid the foundation for utilizing the brain networks of resting-state fMRI for EEG source imaging (see Section Resting-state fMRI).

**Figure 3:**
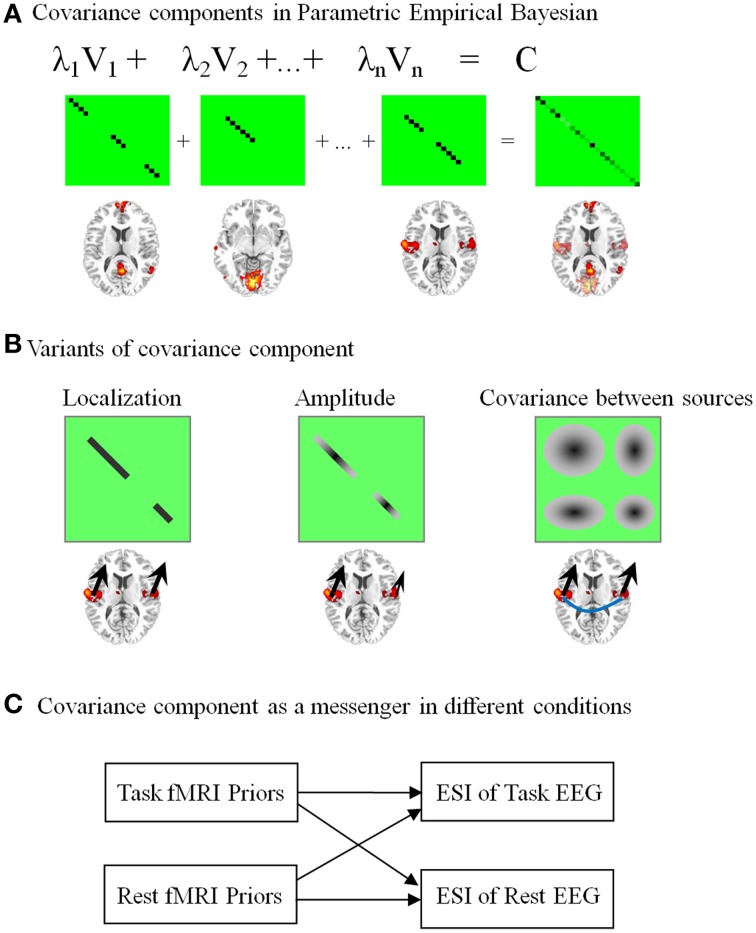
**Network-based source imaging and its variants**. The brain maps represent the spatial pattern of the upper covariance components (CCs). **(A)** Temporally coherent networks derived from fMRI are employed as the CCs in parametric empirical Bayesian model. **(B)** Three different structures of CCs are illustrated: a covariance matrix with binary value only considers the location information from fMRI (the black arrows have the same size); a covariance matrix with continuous value assumes the magnitude of neuroelectric activity based on fMRI statistical quantities (arrows with different size); a covariance matrix with non-zero off-diagonal terms has a strong assumption that EEG sources in a fMRI cluster have coherent time course (the blue line between arrows). **(C)** CCs are messengers to transmit the information between task-evoked and resting-state brain activation.

NESOI contains three steps to reconstruct the EEG source. First, brain functional networks are extracted from fMRI by spatial independent component decomposition. The intensity values in each TCN are scaled to z scores. Second, each spatial pattern (or TCN) is thresholded to form the spatial connected clusters. Voxels with absolute z scores larger than a threshold are considered to show activation. Third, each cluster is then independently projected onto the surface mesh, leading to the definition of a covariance component. A node in the EEG source space is assigned according to the z score of its nearest-neighbor fMRI voxel after spatial registration. All the activated nodes in each TCN showed similar temporal dynamics of the BOLD signals, thus we assumed they had similar properties for EEG signal generation. For the PEB framework of ESI, the simplest way to construct a covariance component (see Equation 3) from a TCN is to assign the diagonal terms by 1.0 on condition that the corresponding node is activated, and it assigns other terms by 0.0. NESOI takes into account the local coherence in source space and introduces a sophisticate covariance component based on TCNs (Figure 3A). Using synthetic and real data, we have proved that incorporating TCNs have significantly facilitated the EEG source estimation when compared with other source inversion methods (Lei et al., 2011b).

The covariance component in our later development has multiple variants (Lei et al., 2012a). As illustrated in Figure 3B, the simple covariance component only contains 0 or 1 in the diagonal terms, i.e., only the information about location from fMRI is used for ESI. In contrast, a covariance component with continuous value considers the fMRI statistical quantities as the amplitude of neuroelectric activity. Finally, a covariance component with non-zero off-diagonal terms could further model the correlated EEG sources. The last model has a strong assumption that EEG sources in a cluster have coherent time course (see the blue line between arrows in Figure 3B). The advantage of using a detailed covariance component is its flexibility for diverse coupling between EEG and fMRI (Lei et al., 2012a). Moreover, mutability of EEG sources across task and subject can also be dealt effectively in this model. As illustrated in Figure 3C, covariance components can even be the messengers to transmit information between task and resting-state brain activity.

### Resting-state fMRI

Brain in resting state is an attractive topic in recent neuroimaging studies (Fox and Raichle, 2007). The term “Resting State” mainly refers to the coherent fluctuations of brain oscillations in different brain regions, while the subject is at rest without any particular stimulus or task. Distinct patterns of coherent activity have been identified in resting-state fMRI. Resting state networks (RSNs) involving predominantly visual areas, auditory areas, sensorimotor areas, and those areas are known to be involved in attentional processes and have been shown to be reproducible across large populations (Smith et al., 2009). Intriguingly, the spatial patterns of resting-state networks closely matched with the task-evoked activation used by the brain undergoing a comprehensive set of task types (Smith et al., 2009). In addition, these resting-state networks are continuously and dynamically “active” to constitute the full repertoire of functional networks utilized by the brain in action. These patterns may facilitate EEG source imaging, not only to EEG rhythm during resting state, but also to event-related potential (Lei et al., 2011b).

Here we expanded the NESOI method above, named resting-state NESOI (rsNESOI), which is modified to include RSNs derived from resting-state fMRI as priors (Lei, 2012). The rsNESOI employs RSNs with fixed spatial patterns; hence it is free from extra fMRI scan to obtain the spatial priors. Templates of nine RSNs constituted multiple spatial priors for ESI. The RSNs were extracted from a resting-state fMRI dataset, which consisted of 42 healthy participants (20 females, age 18–27) from the Southwest University. All the data were mainly pre-processed and were subjected to a group ICA (http://icatb.sourceforge.net/). Then spatial patterns of resting-state fMRI were displayed and explained based on the result of Smith et al. (2009). Nine RSNs were extracted because of their similar spatial distribution with the reported pattern (Smith et al., 2009). The first three resting-state network corresponded to medial, occipital pole, and lateral visual areas. Default mode network was a large structure in the middle frontal, posterior cingulate, and inferior temporal gyrus. Sensorimotor, auditory, and executive control networks were usually utilized by the brain in some special action. Left- and right-lateralized frontoparietal were the only maps to be strongly lateralized. They covered several frontoparietal areas. Networks were then employed as covariance components of PEB for EEG source imaging (Lei, 2012). The main difference between rsNESOI and NESOI is the utilization of RSNs instead of the various functional activation constraints. More importantly, because RSNs is fixed and no need to extract from each experiment, the major novelty of rsNESOI is the utilization of multiple RSNs derived from resting-state fMRI without any fMRI scan (Lei, 2012).

ESI of resting state EEG may have great improvement with priors from resting-state fMRI. It is widely believed that neuronal oscillations are the basic mechanism that defines functioning and interaction within and between the modules of large-scale brain networks, and thus are the basic mechanism of cognitive processing (Buzsáki and Draguhn, 2004). The dynamic electrophysiological activity for rhythmic EEG can be reconstructed on the condition that there is a good degree of spatial concordance of the modules of large-scale functional networks. This source imaging of the spontaneous EEG may provide important findings in the understanding of brain functioning and variations of these functions during rest, sleep, cognitive task, maturation or psychiatric diseases.

## Connectivity priors for ESI

The question of how brain regions communicate with each other is of increasing interest for the EEG community. With its fast recording, EEG is much better suited to study the interactions between brain regions during various cognitive tasks and resting-state (Hillebrand et al., 2012). The dynamic information provided by an EEG-derived network allows for a precise definition of the location and timing of cognitive processes. For example, dynamic changes of the epileptic brain can enhance our understanding of seizure generation, propagation, and termination (Tyvaert et al., 2009). Connectivity analysis directly applied to the scalp electrode is problematic, because of the volume conduction of the brain tissue and the influence of the reference on connectivity measures.

The ambiguous measurement of scalp electrodes can best be solved by assessing connectivity between time series. Those time series are extracted after having done ESI. Two types of method can execute connectivity analysis in source space. One is the generate model based on biophysics. For example, the neural mass model has the potential to simulate the activity of neural ensembles and the connections between them, so that it can generate brain rhythms or event-related responses (David and Friston, 2003). Another is applying the connectivity algorithms to the signals from the source space, i.e., EEG source imaging combining with multivariate connectivity measures determines the connectivity patterns of cortical activity (Astolfi et al., 2007). Although a lot of techniques are available for connectivity inference in cortex space, there are few studies utilizing “connectivity” priors (note the difference from “spatial” priors) from other modalities. First, with EEG's extremely high temporal resolution, there is hardly any technique has higher temporal information than EEG that can be utilized as a “prior” in ESI. Second, the lack of unified model to simultaneous ESI and connectivity analysis prevents developing effective strategy to include connectivity priors. Here we introduced some potential strategies to incorporate connectivity priors in ESI.

### Structural connectivity prior and DTI

Structural connectivity defines the magnitude and time lag of connection between brain regions, which is critical for the long-rang communication. DTI quantifies the magnitude of water diffusion *in vivo* non-invasively and describes three-dimensional fiber tracking in each voxel. Anatomical parcellation based on DTI can be used as a spatial prior for EEG source imaging. The logic behind is that cortex belonging to the same anatomical unit should tend to exhibit similar activity. A recent work incorporated the percellation information derived from diffusion weighted MRI into the reconstruction of distributed source (Knösche et al., 2013). In ESI, inferring cortex connectivity based on coherence or phase lag index is sensitive to indirect connections. The fiber tracking obtained from DTI can be used to define the backbone of the network in the source space. A recent source imaging study imposed stronger penalties to the cortex connection that corresponded to weak anatomical connection (Pineda-Pardo et al., 2014). The expressivity of the source model was improved after introducing anatomical prior from diffusion weighted MRI, leading to a better classification between psychotic patients and healthy controls groups (Pineda-Pardo et al., 2014).

Because information transfer depends on the length of white matters, so the neuroanatomical structure provided by DTI can be further used to determine the interaction delay of the macroscopic brain networks. This is valuable for ESI to estimate the phase relation of brain oscillation. For example, the phase pattern of the oscillatory neuronal activity has been studied with the structural connectivity between regions of the whole brain (Stam and Van Straaten, 2012). One study found that cortical networks derived from source EEG estimates partially reflect both direct and indirect underlying white matter connectivity in all frequency bands evaluated. In addition, functional connectivity was significantly reduced for high frequency bands compared to low frequency bands when structural support is absent (Chu et al., 2015). Intriguingly, without external stimulation, a clear spatial pattern of phase relations emerged with regions belonging to the default mode network. In another study, the white matter architecture precisely determined the dynamic character of EEG rhythm. A positive relation was identified between EEG alpha rhythm and white matter architecture within the posterior and superior corona radiata (Valdes-Hernández et al., 2010).

### Disturbing connectivity with TMS

TMS is a technique that allows for the non-invasive brain stimulation. It alters cortical excitability, effective connectivity, and oscillatory tuning of a given cortical area, and hence provides both spatial and connectivity priors for ESI. TMS delivers a brief high-intensity magnetic pulse to the head through a coil. Electrical currents are induced in a focal area underneath the coil, and they interact with ongoing activity in the neural tissue. These brief currents, producing excitation or inhibition of the stimulated cortical area, can transiently influence potential distribution (Walsh and Cowey, 2000). Spatial priors from TMS in ESI may give priority to superficial areas because the strength from the magnetic field falls off rapidly with distance from the TMS coil. While for the activating areas beneath the coil, cortico-cortical and cortico-subcortical connections can also be tested in the concurrent TMS-EEG. In fact, a neuroimaging study has revealed that TMS also results in activation of remote brain regions connected to the site of stimulation with long-range connections (Rogasch and Fitzgerald, 2013).

### Granger causality analysis

More recently, effective connectivity has been introduced in the EEG community to capture causal relationships within brain circuit. Multivariate methods such as structural equation modeling, partial directed coherence, and directed transfer function are widely employed, although most of these methods are based on the Granger theory of causality (Astolfi et al., 2007).

An acceptable connectivity prior is the causal connection derived from fMRI. Though with relatively low temporal resolution, Granger causality analysis is widely utilized in fMRI for certain brain regions (Roebroeck et al., 2005). If the overlapped activation areas from EEG and fMRI were related to the same neural activity, it is rational to compare the causality inferred from both modalities. In a simultaneous EEG-fMRI study, we compared the causal connectivity derived from EEG and fMRI (Lei et al., 2011a). Interestingly, the links between the same two areas were distinct different for EEG and fMRI. Our data provided a complex relationship between modalities, which should be considered cautiously when applying fMRI connectivity priors in ESI (Lei et al., 2011a).

For resting-state EEG, the connectivity of slow fluctuations of EEG has its natural correspondence to BOLD signals because of the same time scale. In this situation, the connectivity identified by fMRI provides a lag version of the EEG interaction because of the convolving influence of hemodynamic response. Though inter modality connectivity may be useful to compare the results between EEG and fMRI, an interesting analysis may infer the intra modality connectivity from a concurrent EEG/fMRI recording. Based on EEG inverse solution and concurrent BOLD signals, previous study revealed that there was no influence of the fMRI on the EEG but the EEG sources had great influences on the fMRI (Valdes-Sosa et al., 2011). For the slow phenomena of the α-band EEG activity, a consistent causal model of EEG was induced to fMRI modulation.

Though it is possible to consider that the connectivity structure obtained from fMRI can be included as a prior to estimate EEG source connection, it is hard to evaluate it. Because the independence of EEG is attenuated as its result may bias toward the connection of fMRI. In addition, the BOLD correlations are found in very low frequency ranges of < 0.1 Hz. This is much too slow to be relevant for ongoing cognitive activities that change much more rapidly and lead to different spatial patterns of coordinated networks within fractions of seconds. Many studies used a two-step method: source imaging and then compare functional connectivity derived from fMRI and EEG in parallel. Complementary neuroelectric and hemodynamic information are referred here to help explain the complex relationships between different brain regions.

### Dynamic causal modeling

Dynamic causal modeling (DCM) is an analytic tool developed to study directionality and causality relationships between M/EEG or fMRI sources (Friston et al., 2003; David et al., 2006). Initially developed to model the event-related potentials of equivalent current dipole, DCM has recently been applied to spatial distributed source (Daunizeau et al., 2009). It provides a cascade of forward models from neuronal features at the micro-scale to the effective connectivity structure at the macro-scale. DCM of EEG involves two levels of description. At the meso-scale of local neuronal population, neural mass models describe the dynamics of local excitatory and inhibitory subpopulations. At the macro-scale of the effective connectivity structure of brain regions, the forward, backward, and the lateral connection constitute interactions among source units. The EEG signal is estimated by computing the instantaneous electrical potential generated by the pyramidal cells, which has been spread through the head volume.

DCM employs large-scale computational modeling and requires the explicit definition of the neuronal substrates that elicit EEG measurements. On one hand, parameters at the meso-scale, such as the synaptic time constant of excitatory and inhibitory neurons, are motivated by experimental findings typically obtained from animal studies (Friston et al., 2003). In ESI, they have to be constantly adapted to novel experimental insights as the priors. On the other hand, parameters at the macro-scale such as lateral connection may be derived from structure priors of DTI and MRI. Because of its comprehensive model in meso and macro-scale, DCM bridges the gap between our understanding of brain activity at a cellular level and on a whole-brain scale, and it may provide a deeper understanding of the neural mechanisms underlying the mental processes of interest (Deco et al., 2008; Valdes-Sosa et al., 2009a).

The aforementioned DCM of EEG directly utilized priors from animal studies and structure neuroimaging. Additionally, DCM can be applied to fMRI signals, and then the revealed connectivity parameters may be utilized as priors in ESI. An interesting question may be that which connectivity priors derived from fMRI are more accurate, as both DCM and Granger can reveal the connectivity for fMRI signal. Considering the theoretical background, Granger causality, and DCM was based on the original fMRI time series and the hidden state variables, respectively. A previous study compared the results of Granger causality and DCM with the functional coupling estimated from intracerebral EEG (David et al., 2008). The neural driver of spike-and-wave discharges was estimated accurately in DCM. Functional connectivity analysis applied directly on fMRI signals failed because hemodynamics varied between regions, rendering temporal precedence irrelevant. Because DCM determined the connectivity from hidden state variables, it may eliminate the hemodynamic effects, and further provide more accurate connection priors to ESI.

## Future directions

EEG source imaging provides a detailed picture of neural activity in the cortex space, which is helpful to understand the dynamic relationships between brain regions. However, because EEG information alone provides limited spatial resolution, advancing ESI requires the integration of inversion model with other modalities. Below we discussed the current trends of the methodological development in this fast developing field.

### Physiological data and ESI

The wide application of diverse sensors makes the physiological data, such as skin conductance, heart rate, and peripheral signal accessible and available for neuroimaging. The current dipole moment density *q*, defined as the moment of an equivalent current dipole per surface area of active cortex, is an independent parameter of size of active tissue. Recent study revealed that the value of *q* has a maximum value in physiological conditions across brain structures and species (i.e., 1–2 nAm/mm^2^), and this maximum value may serve as an effective physiological constraint for MEG/EEG inverse solutions (Murakami and Okada, 2015). Other physiological measurements are useful in ESI, because they are informed in interpretation of the results. For example, the arousal state of the subject may be used to judge the physiological significance of ESI. Thus, the purpose of multimodal imaging would not be to merge data but to allow informed judgment on the context of the data. The auxiliary data can yield biomarkers to assess the state of the brain tissue, which might account for intra- and inter-subject variability usually not accounted for.

### Relationships between brain rhythms

EEG directly measures synchronized neuronal activity across a wide range of frequencies. The brain rhythms in specific frequency bands have been related to distinct functions. In terms of perception, for instance, γ-band (30–100 Hz) has been linked to perceptual grouping and maintenance in visual memory (Foster and Parvizi, 2012). In contrast, the slower θ (4–8 Hz) and α-band (8–14 Hz) have been related to interregional, long-distance interaction for control of lower-level by higher-order areas or for unification of cognitive operations through phase-coupling with other frequency bands, besides more local processes (Foster and Parvizi, 2012). Intriguingly, oscillatory activities of different frequency bands have been reported to interact with each other in several contexts, suggesting the possibility that different frequency oscillations might carry different dimensions of the integration process (Florin and Baillet, 2015). One important conclusion of a cross-frequency coupling analysis is that the beta rhythm is robust for long-distance synchrony, whereas gamma rhythms tend to be more stable for local patches of synchrony (Foster and Parvizi, 2012). Cross-frequency coupling may be a pervasive mechanism used by the brain to perform the network-level dynamic computations that underlies brain integration. The future extension of ESI needs to consider the cross-talk between frequencies and develop new reconstruction models.

### Combining ESI and network construction

Current method constructs EEG cortex network in two steps, i.e., the source imaging is independent from the connectivity analysis. A unified framework is required to estimate source distribution and coupling parameters simultaneously. DCM provides an extendable framework to incorporate source imaging methods. In DCM of evoked responses, lead field is parameterized with finite precision. It enables the data to inform the network's spatial configuration and its expression at the sensors (Kiebel et al., 2006). Alternatively, discovering connectivity may also be expressed as the state-space models with biophysically informed observation and state equations. These models have to be endowed with priors on unknown parameters and afford checks for model identifiability (Valdes-Sosa et al., 2011). As ESI and connectivity analysis are corresponding to observation and state equation, respectively, a unified framework can be derived from this condition.

### From ESI to multimodal fusion

The spatial constraint in ESI does not consider other modalities equivalently or analyze them jointly. It is a typical asymmetric multimodal integration (Lei et al., 2012b). In contrast, a symmetric integration constructed a common model to explain the EEG and other modalities (Daunizeau et al., 2007; Valdes-Sosa et al., 2009a; Lei et al., 2010). The symmetric integration is widely applied in EEG and MEG fusion. We can further categorize the symmetrical integration into the model-driven and the data-driven integration.

Based on the assumption that EEG and fMRI signals are generated by the same cortical regions with a specific neural population, the model-driven symmetric integration develops biophysical generative models to inverse both EEG and BOLD signals. Datasets of simultaneously acquired signals are entered into a comprehensive model of brain activity, neurovascular coupling, and finally relating BOLD signals and scalp EEG signals to underlying neuronal activity (Daunizeau et al., 2007). The inverse solutions of these models are the neuronal activity and connectivity of the underlying neural population. The symmetrical integration based on a cascade of generation models provides a deeper understanding of the neural mechanisms underlying mental processes of interest (Daunizeau et al., 2007; Valdes-Sosa et al., 2009a).

The model-driven symmetrical integrations employ highly detailed computational modeling and require the explicit definition of the common neuronal substrates that elicit both EEG and fMRI measurements. Recently, we integrated both “temporal prediction” and “spatial constrain” into a single framework, and then obtained a data-driven symmetric integration (Lei et al., 2012b). Spatial temporal EEG/fMRI fusion (STEFF) employs spatial constraint and temporal prediction fusions in parallel. The fMRI spatial patterns are employed as the covariance priors of the EEG source distribution, mean while, the trial-by-trial dynamics extracted from EEG are utilized to form the design matrix of the fMRI time course (Lei et al., 2010).

Any attempt at incorporating prior information from other modalities into ESI inevitably encounters the problem that EEG and the measurements of other modalities may be generated by different physiological processes. Because of the mismatch with each other, there is likely to be a disparity between the activation areas revealed by EEG and other modalities. It is necessary for a fault-tolerant ESI model to incorporate this candidate information. For investigators, conventional approaches may fail because of their tendency to the converging evidence of each modality. In fact, some neural processes are independent between modalities; that is, brain activity may be visible for one modality while blind for the other. Analysis of these model-specific components would be equally important for understanding of various cognitive processes (Lei et al., 2012b).

## Conclusions

Because of its outstanding property in temporal resolution, as well as because it is free of radiation and easy to set up, EEG is used extensively in both neuroscience research and clinical applications. Both the functional and the effective connectivity can be reconstructed in the source space. By introducing reasonable priors from other modalities, EEG source imaging reveals the most probable sources and their related networks at every moment in time. Here, we reviewed the available priors from MRI, fMRI, DTI, PET, TMS, etc. We focused our review on task-evoked activation map derived from fMRI, PET, especially from EEG-correlated fMRI. Then we introduced the priors for connectivity analysis, which included resting-state fMRI, DTI, TMS, the simultaneous EEG-fMRI and EEG-TMS. More than providing definitive answers we aimed to identify the important open issues in the quest of incorporating priors from other modalities in EEG source localization. We suggest that combined EEG source imaging with other complementary modalities, simultaneously detailed the temporal and spatial dimensions of brain activity, making it a promising approach toward the study of neural networks in cognitive and clinical neurosciences.

### Conflict of interest statement

The authors declare that the research was conducted in the absence of any commercial or financial relationships that could be construed as a potential conflict of interest.
